# Modification of biopharmaceutical parameters of flavonoids: a review

**DOI:** 10.3389/fchem.2025.1602967

**Published:** 2025-04-29

**Authors:** Amir Taldaev, Artem A. Svotin, Semyon I. Obukhov, Roman P. Terekhov, Irina A. Selivanova

**Affiliations:** ^1^ Laboratory for the Study of Single Biomacromolecules, Institute of Biomedical Chemistry, Moscow, Russia; ^2^ Laboratory of Biomolecular NMR-Spectroscopy, Shemyakin-Ovchinnikov Institute of Bioorganic Chemistry, Moscow, Russia; ^3^ Research Center for Molecular Mechanisms of Aging and Age-Related Diseases, Moscow Institute of Physics and Technology, Dolgoprudny, Russia; ^4^ Nelyubin Institute of Pharmacy, Sechenov First Moscow State Medical University, Moscow, Russia

**Keywords:** flavonoids, solubility, permeability, bioavailability, biopharmaceutical properties, modification, review

## Abstract

Flavonoids are natural organic compounds that are derivatives of diphenylpropane. This group of polyphenols can be found in multiple natural sources and they exhibit a variety of biological effects. Despite the wide array of beneficial properties, the development of drugs based on these compounds is hindered by their low bioavailability. Although the substantial body of information available on strategies to enhance the solubility and bioavailability of flavonoids, this knowledge remains fragmented. Therefore, the aim of this study was to consolidate and systematize scientific data on methods for increasing the solubility and bioavailability of flavonoid compounds without changing their initial molecular structures. Throughout the investigation, it was determined that the most prevalent methods for increasing solubility and bioavailability include co-crystallization, formation of phospholipid and inclusion complexes, and the creation of nanostructures. Although there were no pronounced differences observed in enhancing solubility, the impact of these methods on pharmacokinetic parameters was established. It was found that the production of inclusion complexes and nanostructures leads to the greatest increase in the area under the pharmacokinetic curve by an average of 4.2 and 3.7 times, respectively. The least effect was noted for phytosomes, where this parameter for the modified forms exceeded the initial value by only 1.7 times. Phospholipid complexes exhibited a longer average half-elimination time than all other modifications, achieving a 2.1-fold increase. For nanostructures and micelles, a substantial increase in maximum concentration of the active substance in blood plasma was observed, reaching an average of 5.4 times for both types of modifications. During the systematization and generalization of the data, a high level of heterogeneity in solubility assessment methods across various studies was revealed, complicating comparisons of original data obtained by different researchers. The findings of this review are crucial for researchers investigating the bioavailability of flavonoid compounds and will facilitate the selection of the most effective methods based on the desired outcomes for solubility and bioavailability.

## 1 Introduction

Flavonoids are natural organic compounds that are derivatives of diphenylpropane. This group of polyphenols can be found in multiple natural sources, including fruits (Ellwood et al., 2019), vegetables (Ahmed and Eun, 2018), berries (Whyte et al., 2019), tea (He et al., 2021), and a large number of medicinal plants (Tungmunnithum et al., 2018; Wang et al., 2018; Roy et al., 2022). Flavonoids exhibit a variety of biological effects, including anti-hepatotoxic (Gul et al., 2022), anti-ulcer (Zhang et al., 2020), and anti-inflammatory activities (Maleki et al., 2019; Al-Khayri et al., 2022), as well as wound healing properties (Carvalho et al., 2021; Zulkefli et al., 2023; Svotin et al., 2025). Several researchers claim that the anti-inflammatory effects of certain flavonoids result from the inhibition of interleukins (IL-1β, IL-6 and IL-8) and tumor necrosis factor TNF-α (Zaragozá et al., 2020). Possible mechanisms for the wound healing activity of flavonoids include their involvement in the regulation of the MAPKs and NF-kB signaling pathways (Lu et al., 2021; Ding et al., 2023). Flavonoid-related compounds, specifically isocoumarins, have been shown to exhibit anti-inflammatory activity by inhibiting enzymes involved in the leukotriene and prostaglandin pathways (Ramanan et al., 2016). Additionally, they may modulate neuronal functions through interaction with the neurotrophin receptor TrkB (Sudarshan et al., 2019). Furthermore, these compounds are potent antioxidants, capable of trapping free radicals (Masuoka et al., 2012; Tumilaar et al., 2024).

Many flavonoids are optically active compounds due to the presence of chiral carbon atoms in the benzopyranone ring. However, most researchers do not adequately address the issue of the stereochemistry of these substances. Nevertheless, this factor can lead to variations in the physicochemical, pharmacokinetic, pharmacodynamic, and pharmacological properties of various active pharmaceutical ingredients (APIs). Some scientists suggest that the lack of data regarding the stereochemistry of flavonoids may contribute to incomplete information about their safety and efficacy (Terekhov et al., 2024).

Despite the wide array of beneficial properties exhibited by flavonoids, the development of drugs based on these compounds is hindered by their low bioavailability. According to biopharmaceutical classification system (Charalabidis et al., 2019), bioavailability is influenced by the solubility of the compound in water and its permeability through the cell membrane. Most flavonoids demonstrate poor solubility in water at room temperature, which limits their bioavailability. In light of their pronounced biological effects, this limitation raises the important issue of how to enhance the solubility of this group of compounds.

**FIGURE 1:**
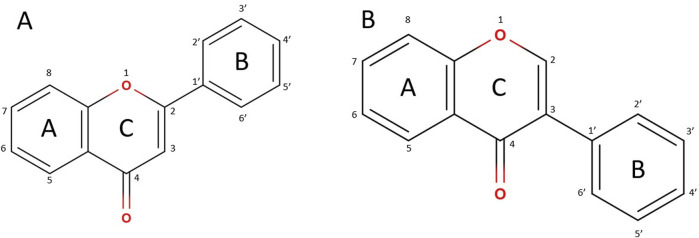
General structure: **(A)** flavonoids; **(B)** isoflavonoids.

An extensive search for methods to enhance the solubility and bioavailability of flavonoids is essential. Currently, variations in solubility in both polar and non-polar solvents are reported, depending on the specific structure of the flavonoid. The presence of a double bond in the ring C (Figures 1A, B), influences solubility, which is further affected by the number of hydroxyl groups in ring B. Additionally, the position of ring B within the benzopyranone structure plays a critical role. When methoxy groups are present in ring B, a decrease in flavonoid solubility is observed, regardless of the solvent used. Conversely, the existence of a single bond between the C2 and C3 atoms in ring C contributes to increased overall solubility, while the introduction of an OH group at C3 reduces solubility in water (Zhang H. et al., 2017). It is important to note that modifications in the chemical structure of these compounds may correlate with changes in biological activity. Consequently, there has been a growing interest among researchers in exploring strategies to enhance solubility without changing the original structure of flavonoids.

In addition to the physico-chemical properties of flavonoids, their bioavailability is also influenced by various other factors, including the dosage form of the drug (Stielow et al., 2023), physiological conditions (Dima et al., 2024), intestinal enzymes (Dima et al., 2020) and microflora (Kan et al., 2022). Moreover, low concentrations of flavonoids in blood plasma and their affinity for albumin hinder absorption (Naeem et al., 2022).

Despite the substantial body of information available on strategies to enhance the solubility and bioavailability of flavonoids, this knowledge remains fragmented. Therefore, it is crucial to systematize scientific information in this domain to identify new avenues for future pharmaceutical development.

The aim of this study was to consolidate and systematize scientific data on methods for increasing the solubility and bioavailability of flavonoid compounds without changing their initial molecular structures. Throughout this work, the main research question is to identify the most commonly encountered methods and evaluate their effectiveness in enhancing bioavailability by improving the solubility and permeability of the original flavonoids.

## 2 Methods

### 2.1 Search strategy

The following review was performed in accordance with the Preferred Reporting Items for Systematic Reviews and Meta-Analyses (PRISMA) guidelines (Page et al., 2021). To perform the literature search, the Google Scholar database was used. The following terms were applied: “flavonoid AND (solubility OR permeability) AND -review”. The search was conducted on publications published no earlier than 2010.

### 2.2 Data processing

Two reviewers (AS and SO) independently and simultaneously performed an initial search and screening of articles by reading their tittles and abstracts to form the reference list. In case of disagreements, they were resolved by another author (RT).

Then, two authors (AS and SO) performed the data extraction of main texts, tables, figures, and Supplementary Material from the selected articles. The following data were in focus of the reviewers: method which used to increase bioavailability, initial and resulting solubility in water, multiplicity of solubility increase, initial and resulting apparent permeability, multiplicity of permeability increase, and main pharmacokinetic parameters. The sum of extracted outcomes was placed in Google Sheets. A complete consensus in the accumulated data was reached without further disagreements.

The result of the systematic analysis is presented as narrative synthesis.

## 3 Results

### 3.1 General outlook on scientific landscape

**FIGURE 2:**
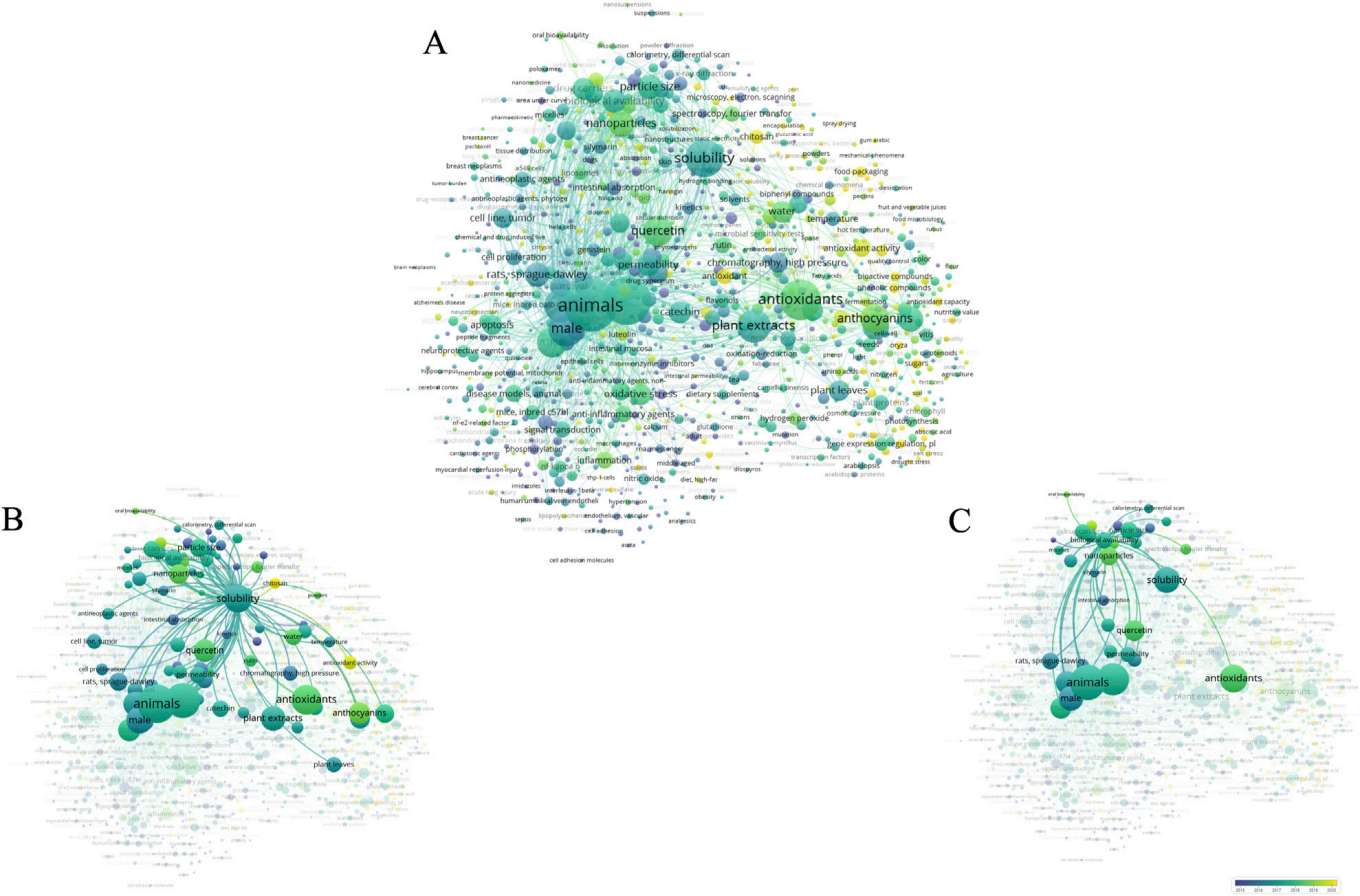
Bibliometric network on request “flavonoid AND (solubility or permeability) NOT review” in PubMed: **(A)** general view of network; **(B)** co-occurrence terms for “solubility”; **(C)** co-occurrence terms for “biological availability”. Created by VOSviewer (Van Eck and Waltman, 2010; Van Eck and Waltman, 2011; Waltman and Van Eck, 2013).

The literature indexed in MEDLINE was utilized to construct a bibliometric network based on query “flavonoid AND (solubility OR permeability) AND (NOT review)” in PubMed (Figure 2A). The term “flavonoid” was excluded from the network.

The size of the bubbles corresponds to the frequency of term mentions. The pseudocolor scale, ranging from blue to yellow reflects the novelty of articles from 2010 to 2024. It is evident that the primary connection to flavonoids is with the terms “animals” and “solubility”. Recently, research in this area has increasingly focused on the study of antioxidant properties (Yang et al., 2024), regulation of gene expression (Bai et al., 2024; Parafati et al., 2024), and chitosan (Liu et al., 2025).

Figure 2B illustrates the interest in flavonoid solubility and related terms. It is apparent that scientists have recently shown a growing interest in the possibility of obtaining nanoparticles derived from flavonoids. Concurrently, considerable attention is given to plant extracts and quercetin as an individual component. Moreover, studies are being conducted on the antioxidant activity associated with solubility.

Figure 2C depicts the relationship between bioavailability and related terms. Over the past 6 years, connections have been observed between bioavailability and nanoparticles (Wahnou et al., 2024), indicating a strong interest among researchers in developing similar structures based on flavonoids. An important aspect is the study of oral bioavailability of flavonoids.

### 3.2 Process of collection and selection of the studies

**FIGURE 3:**
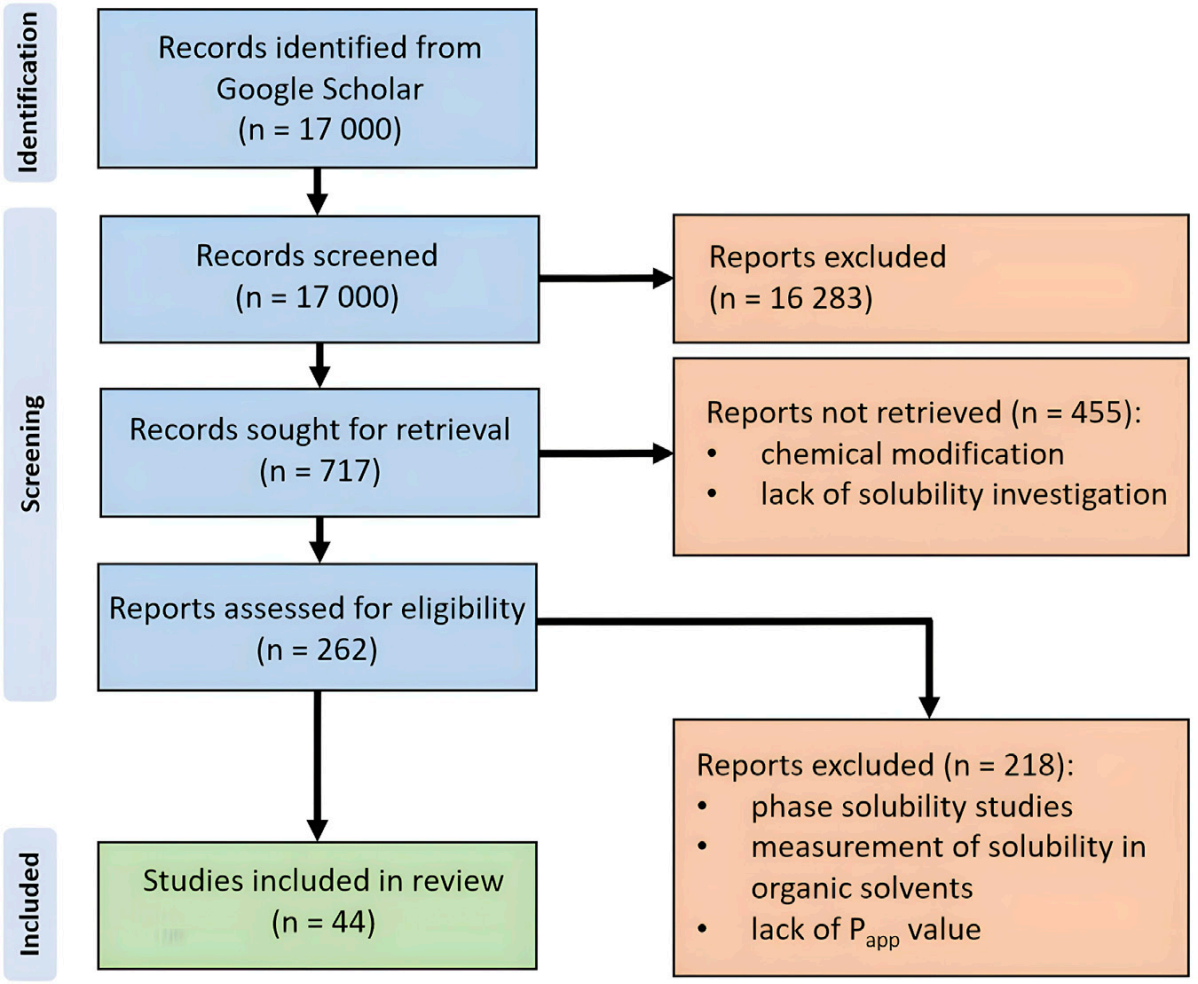
PRISMA flowchart of the search and selection process of the articles.

The initial results of the search identified 17,000 articles in Google Scholar. After the first screening, 16,283 articles were excluded because they did not meet the inclusion criteria based on their titles and abstracts. In the subsequent review, 455 articles were eliminated for the following reasons: chemical modifications of flavonoids that resulted in the formation of new covalent bonds and the absence of solubility investigations. After reviewing the full texts, an additional 218 articles were excluded for the following reasons: reliance on phase solubility studies, measurement of solubility in organic solvents, and a lack of apparent permeability (P_app_) values when the study was associated with permeability evaluation. Consequently, the review included 44 articles that passed through all stages of selection. The collection and selection process is illustrated in the PRISMA flow diagram (Figure 3).

### 3.3 General overview of the included articles

**FIGURE 4:**
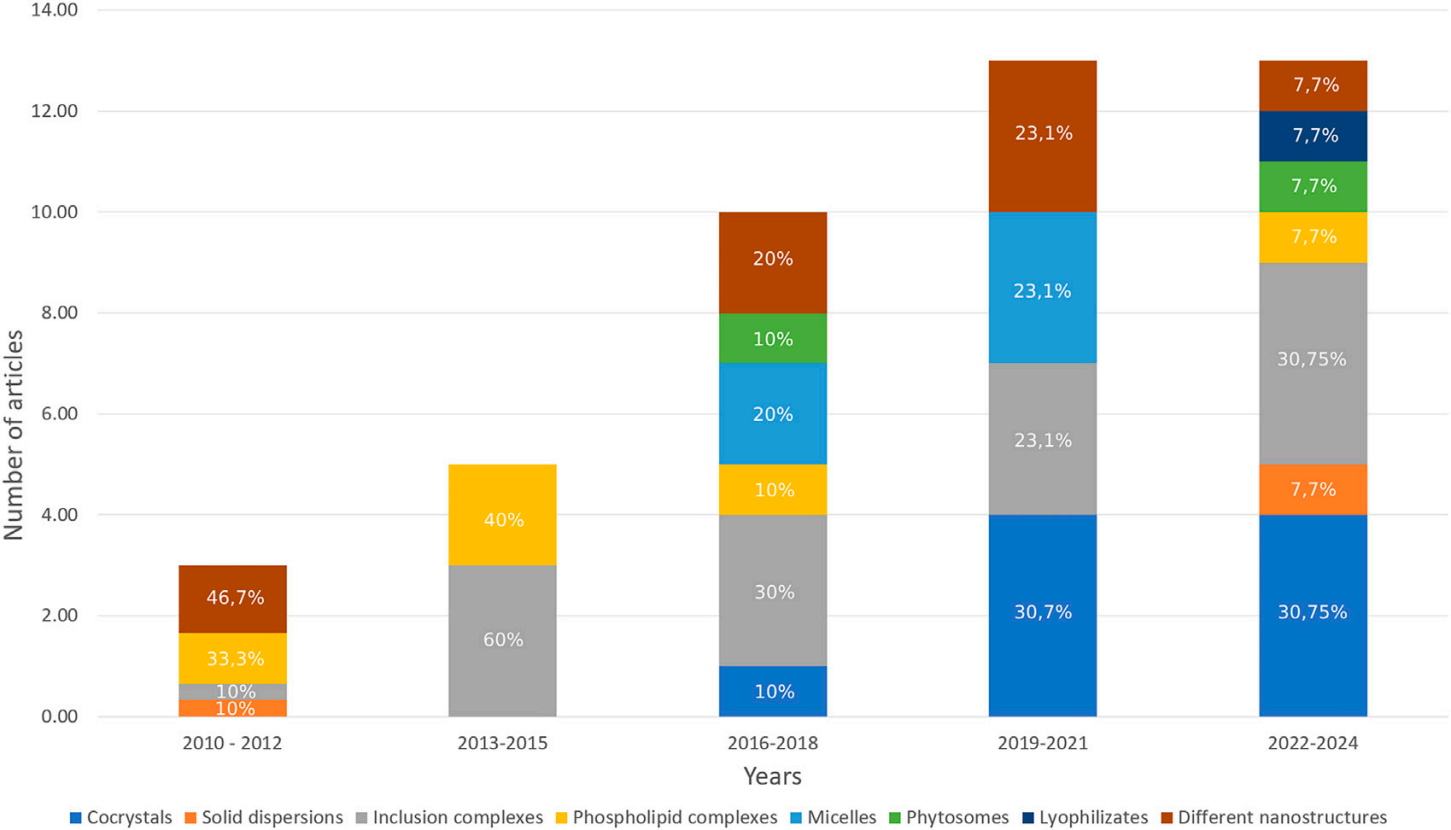
Methods used to increase the bioavailability of flavonoids included in this systematic review (% shows the proportion of the total in a given column).

Since 2010, there has been a steady increase in the number of publications on this topic, rising from 3 in the period of 2010–2012 to 13 in both 2019–2021 and 2022–2024 (Figure 4). During the 2010–2012 timeframe, the primary focus was on the synthesis of various nanostructures; however, by 2022–2024, their representation within the total number of publications has considerable decreased. Additionally, there has been a notable rise in publications on the synthesis of cocrystals, increasing from 10.00% in 2016–2018 to 30.75% in 2022–2024. Inclusion complexes have garnered substantial attention from researchers since the period of 2013–2015. Over the past 6 years, there has been a decline in the proportion of phospholipid complexes among the methods aimed at enhancing the bioavailability of flavonoids. Recently, an increase in the diversity of modification techniques has been observed.

**FIGURE 5:**
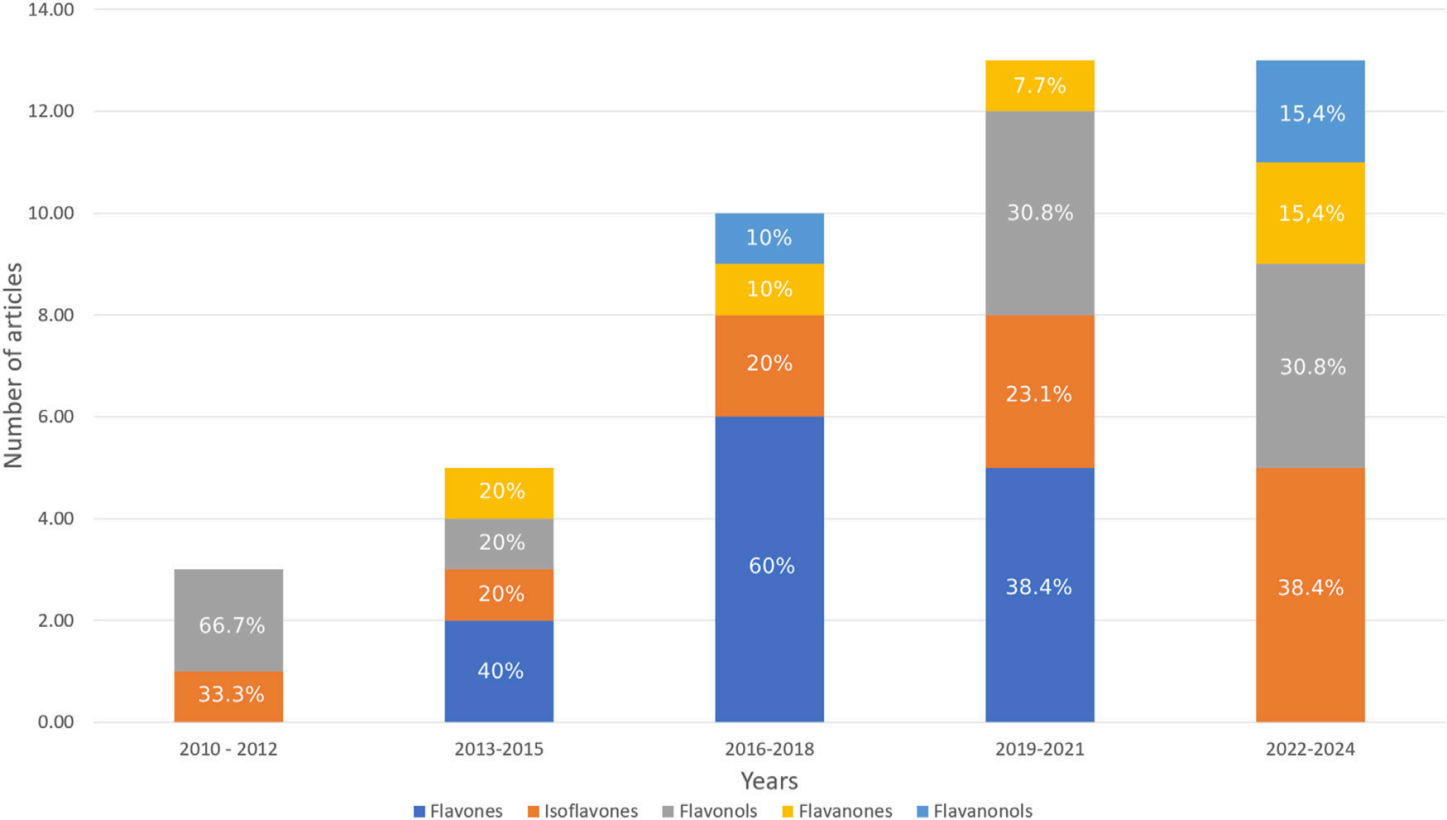
Flavonoids groups included in this review (% shows the proportion of the total in a given column).

**FIGURE 6:**
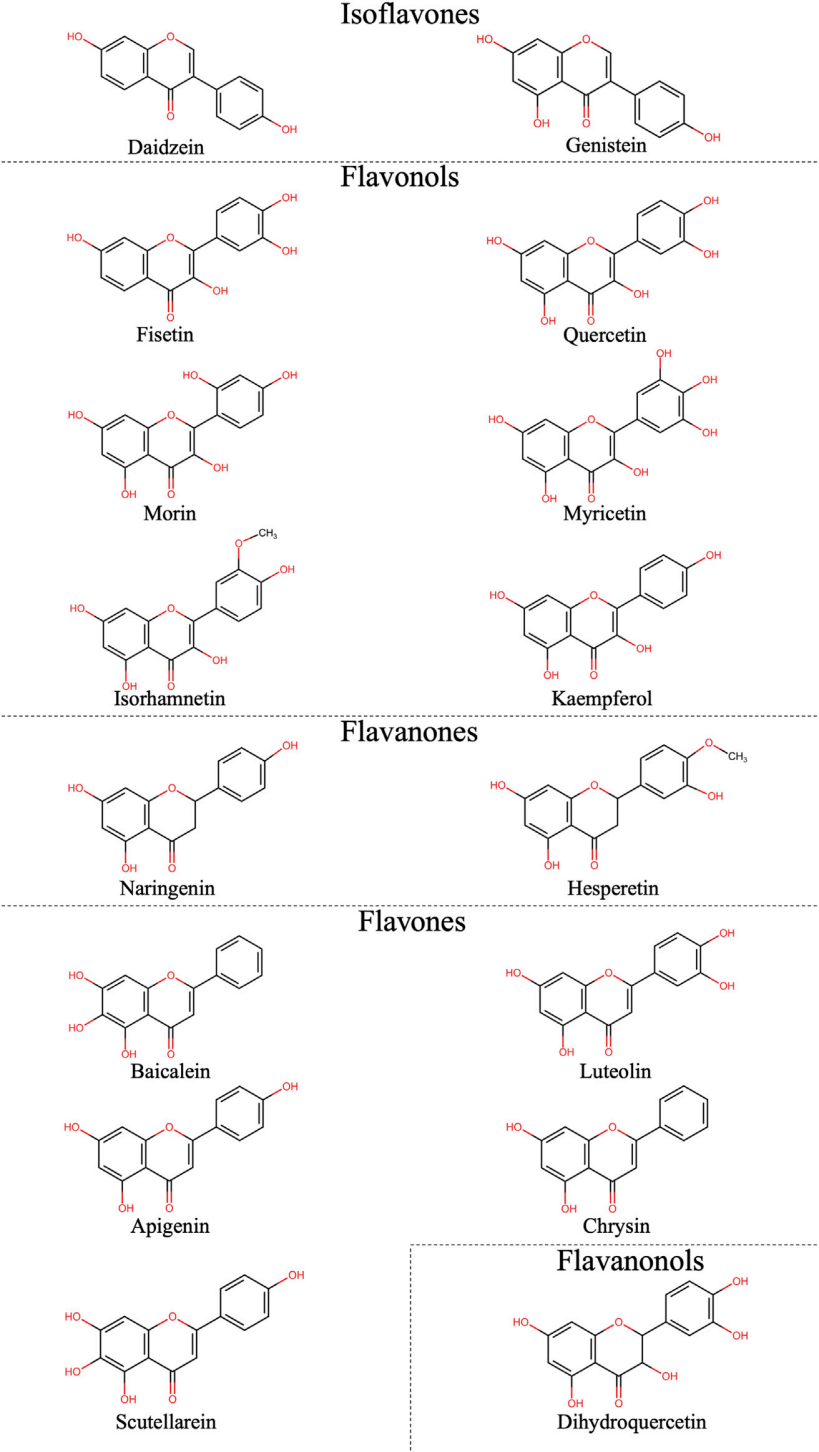
Structures of flavonoids from included articles.

Among the primary groups of flavonoids, isoflavones have attracted considerable attention year after year, comprising 38.40% of all modified structures in 2022–2024 (Figure 5). Although there was a surge of interest in flavones during 2013–2021, no information regarding their modification was identified for the period of 2022–2024. Over the last decade, a greater diversity of flavonoid groups has emerged compared to previous years. Moreover, 15 different flavonoids were identified in the selected articles (Figure 6).

### 3.4 Qualitative synthesis

**FIGURE 7:**
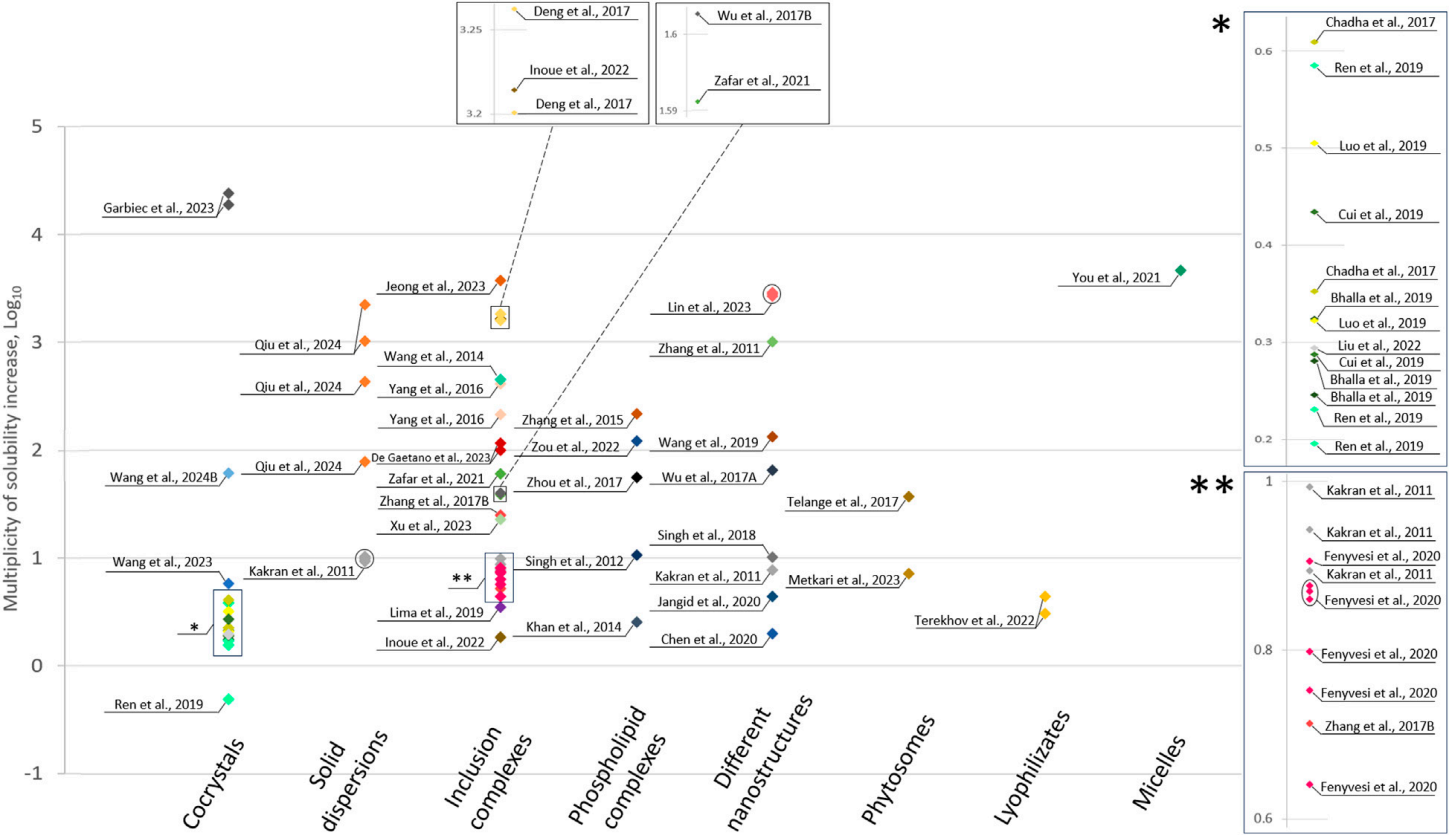
Multiplicity of solubility increase (each color shows a separate publication).

The solubility of the modified objects received attention in 39 articles. Seventy-five distinct modifications were described. For the convenience of graphically representing this data, log_10_ of the multiplicative increase in the solubility of the obtained objects was used (Figure 7). All original solubility data are presented in the Supplementary Table S1.

Nine articles described and characterized 18 new flavonoid cocrystals with improved water solubility. The primary co-formers were nitrogen-containing heterocyclic compounds, including piperazine (Wang et al., 2023; Wang et al., 2024 Z.), nicotinamide (Cui et al., 2019; Ren et al., 2019), isonicotinamide (Bhalla et al., 2019; Ren et al., 2019), theobromine (Bhalla et al., 2019), cytosine (Chadha et al., 2017; Bhalla et al., 2019), caffeine (Cui et al., 2019; Luo et al., 2019; Ren et al., 2019), piperine (Liu et al., 2022), isoniazid (Luo et al., 2019), and thiamine (Chadha et al., 2017). Some cocrystals were formed by combining flavonoids with amino acids such as lysine (Garbiec et al., 2023), arginine (Garbiec et al., 2023), and proline (Ren et al., 2019). The greatest increase in solubility was observed for cocrystals of genistein with proteinogenic amino acids lysine and arginine (Garbiec et al., 2023). In contrast, myricetin cocrystals with caffeine exhibited a 2-fold decrease of solubility in pure water (Ren et al., 2019).

Two articles described solid dispersions based on the flavonoids genistein (Qiu et al., 2024) and quercetin (Kakran et al., 2011) with the addition of polyvinylpyrrolidone or pluronic^®^ F127. Qiu et al. (2024) achieved a solubility increase of over 2000 times for a 1:9 (w/w) dispersion.

The greatest number of solubility modifications (27) was represented by inclusion complexes, as detailed in 13 articles. These modifications primary involved various cyclodextrins and their derivatives, including (2-hydroxypropyl)-β-cyclodextrin (Wang et al., 2014; Yang et al., 2016; Wu et al., 2017b; Lima et al., 2019; Fenyvesi et al., 2020; Zafar et al., 2021; De Gaetano et al., 2023), γ-cyclodextrin (Inoue et al., 2022), amino-modified β-cyclodextrin (Deng et al., 2017), β-cyclodextrin (Kakran et al., 2011; Yang et al., 2016; Zhang Y. et al., 2017; Fenyvesi et al., 2020; Xu et al., 2023), sulfobutylether-β-cyclodextrin (Fenyvesi et al., 2020; De Gaetano et al., 2023), and random methyl-β-cyclodextrin (Fenyvesi et al., 2020). Additionally, some modifications were achieving using cycloamylose (Jeong et al., 2023) and lecithin (Zhang Y. et al., 2017).

Five articles reported the preparation of new flavonoid-phospholipid complexes with improved water solubility. The most notable increase was observed with kaempferol, which achieved a remarkable solubility enhancement of 216.70-fold (Zhang et al., 2015). A slightly lower increase was observed for isorhamnetin (Zou et al., 2022) and baicalein (Zhou et al., 2017), with enhancements of 122-fold and 55.45-fold, respectively. Additionally, a substantial increase (10.70 times) was noted for the phospholipid complex of quercetin (Singh et al., 2012). The smallest increase (2.54 fold) was demonstrated by the modification of luteolin (Khan et al., 2014).

Nanostructures were presented by 10 modifications in eight articles. The following objects were obtained: daidzein–phospholipid complex loaded into lipid nanocarriers (Zhang et al., 2011); nanoparticles with fisetin (Chen et al., 2020), quercetin (Kakran et al., 2011), luteolin (Wang et al., 2019), and apigenin (Wu et al., 2017a); nanosuspension with morin (Jangid et al., 2020) and naringenin (Singh et al., 2018); nanofibers of myricetin (Lin et al., 2023). The greatest increase in water solubility (more than 2600-fold) was exhibited by myricetin nanofibers produced with the addition of (2-hydroxypropyl)-β-cyclodextrin and polyvinylpyrrolidone in various ratios (Lin et al., 2023).

The enhancement of solubility through the formation of phytosomes were addressed in 2 publications. The authors (Metkari et al., 2023) successfully increased the solubility of naringenin by 7.16 times using a fraction of non-GMO soybean lecithin enriched with phosphatidylcholine (LS-75). Additionally, phytosomes containing apigenin (Telange et al., 2017) were created, demonstrating a 36.77-fold increase in solubility.

The only mention regarding an enhancement in solubility involved the lyophilization of a flavonoid solution derived from organic solvents (Terekhov et al., 2022). The flavanonol dihydroquercetin exhibited water solubility increases of 4.41-fold for the aqueous ethanol and 3.06-fold for the acetonitrile lyophilizates, respectively.

A single article detailed an increase in solubility achieved by forming baicalein micelles with glycyrrhizic acid (You et al., 2021). The resulting micelles demonstrated a 4606-fold increase in solubility.

The permeability of the obtained modifications was assessed using Caco-2 cell culture in 7 articles, while 1 publication described cell-free permeation model. In 3 of these studies, the permeability assessment was conducted alongside solubility studies, while in 5 of them it was carried out separately. However, permeability studies were conducted only for two classes of flavonoids: flavones and isoflavones. Micelles (Zhang Z. et al., 2017; Shen et al., 2018; 2019; Ding et al., 2019) and inclusion complexes (Daruházi et al., 2013; Fenyvesi et al., 2020) were most frequently studied modifications in terms of permeability. The permeability of cocrystals (Garbiec et al., 2023) and phospholipid complexes (Zhou et al., 2017) was reported only once each. Most modifications increased permeability by no more than 2-folds, but the inclusion complex with daidzein (Daruházi et al., 2013) achieved 31.40-fold increase (Table 1).

**TABLE 1:** Permeability of modified and native forms of flavonoids.

Group of flavonoids	Compound	Method of increasing permeability	Permeability model	Initial apparent permeability, ×10^–6^ cm/s	Resulting apparent permeability, ×10^–6^ cm/s	Multiplicity of permeability increase	References
Isoflavones	Genistein (GEN)	Micelle formation	Caco-2	6.33 ± 0.49 (AP-BL)	7.82 ± 0.38	1.24^a^	Shen et al. (2018)
				8.65 ± 0.51 (BL-AP)	8.29 ± 0.44	0.96^a^	
		Micelle formation (GEN-F – 1.2 mg/mL; GEN-L – 1.6 mg/mL)	Caco-2	5.28 ± 0.49 (AP-BL)	8.23 ± 0.35 (GEN-L)	1.56	Ding et al. (2019)
				7.97 ± 0.36 (BL-AP)	8.46 ± 0.43 (GEN-F)	1.06	
					7.59 ± 0.56 (GEN-L)	0.95	
		Cocrystallization with lysine (LYS) and arginine (ARG)	Cell-free permeation model	4.28 ± 0.95	0.90 ± 0.02 (GEN – LYS)	0.21^a^	Garbiec et al. (2023)
					1.13 ± 0.03 (GEN – ARG)	0.26^a^	
		Inclusion Complex with RAMEB, HP-β-CD, β-CD, and γ-CD	Caco-2	1.70 ± 0.10	17.10 ± 3.70 (GEN – β-CD)	10.00^a^	Daruházi et al. (2013)
					17.10 ± 3.50 (GEN – HP-β-CD)	10.00^a^	
					6.50 ± 1.70 (GEN – RAMEB)	3.82^a^	
					28.50 ± 1.70 (GEN – γ-CD)	16.76^a^	
	Daidzein (DDZ)	Inclusion Complex with RAMEB, HP-β-CD, β-CD, and γ-CD	Caco-2	11.90 ± 1.90	31.40 ± 4.10 (DDZ – β-CD)	2.64^a^	Daruházi et al. (2013)
					21.70 ± 9.10 (DDZ – HP-β-CD)	1.82^a^	
					24.30 ± 7.30 (DDZ – RAMEB)	2.04^a^	
					16.20 ± 1.30 (DDZ – γ-CD)	1.36^a^	
Flavones	Baicalein	Micelle formation	Caco-2	1.05 ± 0.08 (AP-BL)	1.93 ± 0.19	1.84^a^	Shen et al. (2019)
				0.97 ± 0.10 (BL-AP)	1.85 ± 0.13	1.91^a^	
		Phospholipid complex (BaPC), matrix dispersion based on phospholipid complex (BaPC-MD)	Caco-2	10.1	11.60 (BaPC)	1.15^a^	Zhou et al. (2017)
					15.30 (BaPC-MD)	1.51^a^	
	Apigenin	Micelle formation	Caco-2	5.32 ± 0.51	6.76 ± 0.56	1.27	Zhang et al. (2017c)
	Chrysin	Inclusion Complex RAMEB	Caco-2	2.32	4.65 (1:1 with RAMEB)	2.00^a^	Fenyvesi et al. (2020)
					11.00 (1:2 with RAMEB)	4.74^a^	

^a^ values were calculated by the authors of current review; (CD, cyclodextrin; RAMEB, random methyl-β-cyclodextrin; HP-β-CD, 2-hydroxypropyl beta-cyclodextrin; AP, apical side; BL, basolateral side).

**FIGURE 8:**
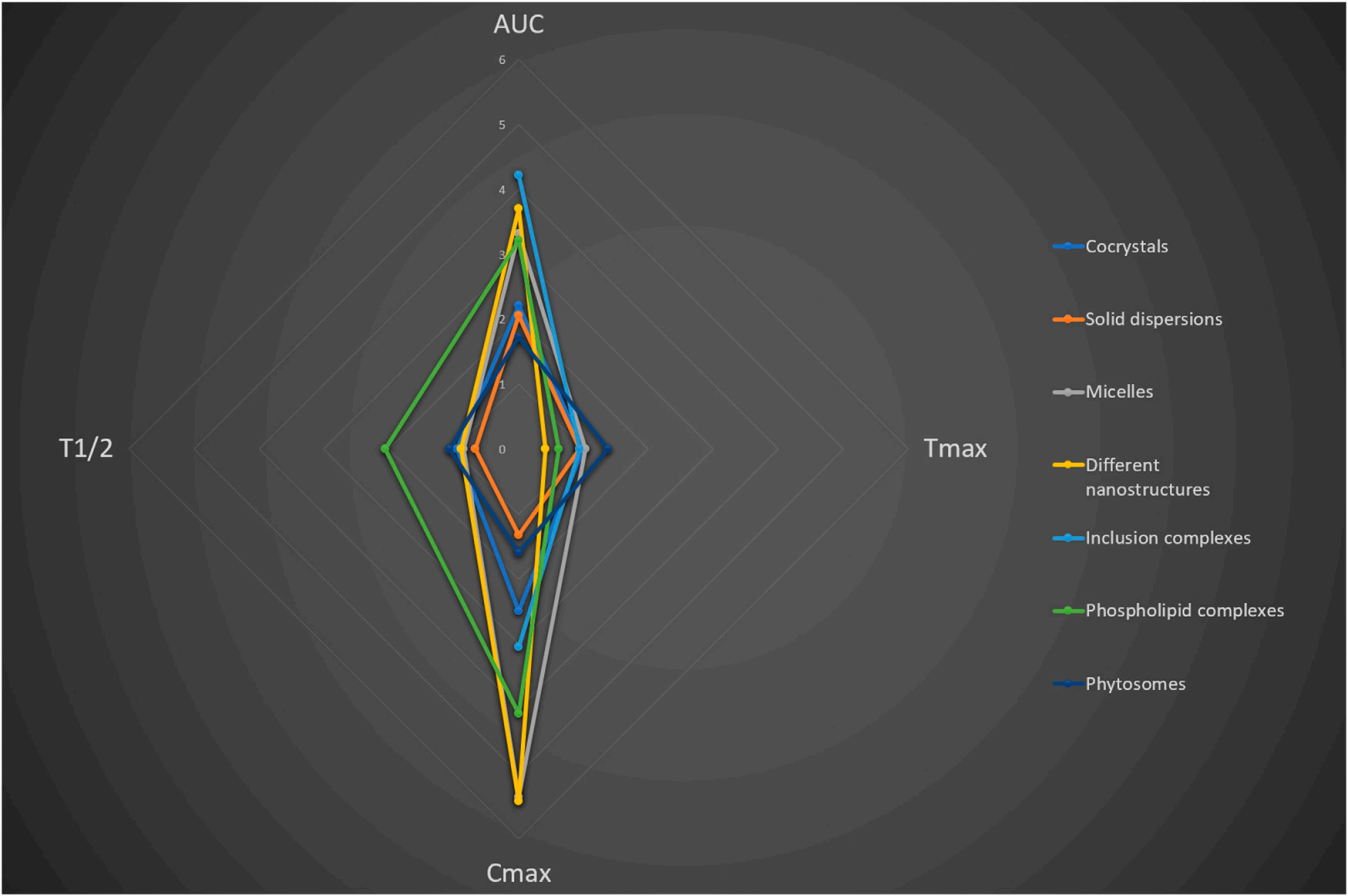
The average multiplicity of the main pharmacokinetic parameters of modified forms in comparison with native flavonoids.

Pharmacokinetic data were also extracted from articles that reported on the solubility or permeability of modifications, when available. Four primary pharmacokinetic parameters were analyzed: the area under the pharmacokinetic curve (AUC), maximum concentration of the active substance in blood plasma (C_max_), time to reach maximum concentration (T_max_) and half-elimination time (T_1/2_). All modifications demonstrated an increased AUC (Figure 8). The greatest improvements in this parameter were observed in inclusion complexes and disassembled nanostructures, with average increases of 4.2 and 3.7 times, respectively. The least effect was noted for phytosomes, where the AUC of the modified forms exceeded the initial value by only 1.7 times. For nanostructures and micelles, a substantial increase in C_max_ of the active substance was observed, reaching an average of 5.4 times for both types of modifications. The production of solid dispersions and phytosomes exerted the least influence on this parameter, resulting in increases of 1.3 and 1.6 times, respectively. Additionally, phospholipid complexes exhibited a longer average T_1/2_ than all other modifications, achieving a 2.1-fold increase. The remaining methods did not show a consistent effect on this pharmacokinetic parameter. None of the methods studied had a notable effect on T_max_, although a slight increase was observed for phytosomes (1.4 times). A decrease in this parameter was recorded for nanostructures and phospholipid complexes, with reductions of 0.4 and 0.6 times relative to the initial time, respectively. All original pharmacokinetic data are presented in the Supplementary Table S2.

## 4 Discussion

A comprehensive analysis of the scientific landscape enabled the identification of several trends regarding the modification of the biopharmaceutical properties of flavonoids in recent years. Considerable attention has been paid to their solubility, as some scientists assert that low solubility in water at room temperature constitutes one of the main obstacles to their application in pharmaceutical practice. Additionally, the objects generated through these modifications are characterized by a range of physicochemical and biological analytical methods. This allows not only for the evaluation of their structures but also for the acquisition of data regarding their bioavailability. Another important aspect is the strong connection to the term “antioxidants”, because natural polyphenolic compounds are natural inhibitors of free radicals. Consequently, they may be utilized in the treatment of oxidative stress (Dhas and Mehta, 2021; Hassan et al., 2022), an area that has recently garnered considerable attention in the scientific community.

The cocrystallization method has demonstrated a considerable positive effect in enhancing the bioavailability of flavonoids. Combining ease of implementation with effectiveness in increasing water solubility, it is regarded as one of the most promising avenues for further investigation (Dutt et al., 2020). Several scientists propose that the increased solubility of these structures, compared to the initial substances, is attributable to the “spring and parachute” phenomenon (Bavishi and Borkhataria, 2016). The resulting cocrystals are supramolecular heterosynthons formed through non-covalent interactions among the components (Raheem Thayyil et al., 2020). This method is considered one of the most promising for enhancing the solubility and bioavailability of oral drugs (Emami et al., 2018; Li et al., 2021; Chhatbar et al., 2025). It is widely employed in modifying the biopharmaceutical properties of polyphenolic compounds, not only in the pharmaceutical industry (Dal Magro et al., 2021; Bu et al., 2023b; Lee et al., 2023), but also in the food industry (Chezanoglou and Goula, 2021; Dias et al., 2021; Irigoiti et al., 2021). Some studies have utilized the cocrystallization process for the subsequent joint separation of a racemic compound mixture (Zhou et al., 2021) and for enhancing the stability of moisture-sensitive drugs (Dhondale et al., 2023). Interestingly, there is a case in which this modification resulted not in an increase, but rather in a decrease in solubility (Ren et al., 2019). The authors attribute this effect to the enhanced stability of myricetin cocrystals with caffeine, resulting from the low solubility of the coformer. A similar decrease in solubility was observed for compounds of other chemical classes, such as the antifungal drug 5-fluorocytosine. (Bu et al., 2023a). In the case of using ferulic acid as a coformer, a decrease in solubility was noted due to the formation of a rigid S-shaped module, which possesses hydrophobic properties and reduced polarity. Other factors that influence the solubility of cocrystals include lattice energy and solvation free energy. For instance, in the case of the matrine modification (Wang Y. et al., 2024), it has been demonstrated that as solvation free energy increases, the solubility of cocrystals in water decreases. When assessing pharmacokinetics, it has been established that cocrystals can significantly increase the AUC and C_max_, while having minimal impact on T_1/2_ and T_max_. This makes the obtained compounds particularly interesting when there is a need to enhance the bioavailability of a drug without affecting the rate of onset of the therapeutic effect or its elimination from the body. Compared to other methods, cocrystallization results in a relatively modest increase in solubility, typically not exceeding 10 fold. However, cocrystallization has successfully produced compounds with the most pronounced increases in solubility when compared to the original compounds, particularly in the case of genistein cocrystals with lysine. Regarding its impact on pharmacokinetic parameters, cocrystallization demonstrates a moderate effect on the enhancement of AUC and C_max_ compared to other methods.

The solid dispersion method is extensively employed in the pharmaceutical industry to improve the solubility and bioavailability of APIs (Tran et al., 2019; Gaber et al., 2022; Bajaj et al., 2023), although it has proven unpopular for flavonoids (only 2 articles). This method has found broad application in modifying the biopharmaceutical parameters of APIs (Bhujbal et al., 2021; Rusdin et al., 2024). During the development of the final product, it is possible to reduce particle size and agglomeration, enhance wettability, and alter the physical state of the substance (Janssens and Van Den Mooter, 2009). These improvements potentially lead to enhanced solubility and bioavailability. There are two primary approaches to obtaining solid dispersions: solvent evaporation-based and melting-based methods. The former includes techniques such as spray drying (Singh and Van Den Mooter, 2016; Ha et al., 2021; Weecharangsan and Lee, 2024), electrospraying (J. Hogan and Biswas, 2008), fluidized bed technology (Kwon et al., 2019), supercritical fluids (Qi et al., 2015), and spray-freeze-drying (Leuenberger, 2002). Melting-based methods comprise hot-melt extrusion (Patil et al., 2016; Gusev et al., 2022; Terenteva et al., 2024), KinetiSol^®^ (Hughey et al., 2012), three-dimensional (3D) printing (Awad et al., 2019), and microwave heating (Hempel et al., 2020). Among these, the most well-known and widely utilized in laboratory and industrial settings are spray drying and hot-melt extrusion. Despite the substantial increase in solubility achieved through the solid dispersion method, its impact on the overall bioavailability of flavonoids was not particularly pronounced. The AUC increased on average 2-fold compared to the initial compounds. This was accompanied by a slight decrease in the T_1/2_ and lack of influence on C_max_ and T_max_. These data indicate the potential of this method in cases where it is necessary to accelerate the onset of a drug’s therapeutic effect while simultaneously enhancing its bioavailability. Compared to other methods, the production of solid dispersions has a limited impact on the primary pharmacokinetic parameters, with the exception of T_1/2_, which shows a reduction.

Interestingly, the method of producing inclusion complexes has gained widespread acceptance as a means to improve the solubility and bioavailability of flavonoids. These structures are formed with cyclic oligosaccharides such as cyclodextrins (CDs), cycloamylose, and its modifications (Allahyari et al., 2025). The ability of CDs to enhance solubility is attributed to their dual structure. Their internal cavity is lipophilic and capable of encapsulating molecules that are poorly soluble in polar solvents through the formation of hydrogen bonds. Meanwhile, the external surface is hydrophilic, ensuring the water solubility of the resulting complexes (Gandhi et al., 2020). Methods for obtaining such structures include co-precipitation (Hoque et al., 2022; Betlejewska-Kielak et al., 2023), kneading (Martins et al., 2020), the supercritical carbon dioxide method (Antipova et al., 2024), grinding (Hoque et al., 2022), microwave irradiation (Bin Jardan et al., 2023), and spray drying (Imam et al., 2022; Soares et al., 2023). Each method is characterized by its own technology, advantages and disadvantages (Cid-Samamed et al., 2022; Drannikov et al., 2022). For flavonoids, this approach significantly enhanced both water solubility and permeability through cellular membranes. This led to the fact that the AUC for inclusion complexes of flavonoids increased on average by 4.2 times compared to the initial substances. This was accompanied by an increase in C_max_ by approximately 3 times. These values represent the highest enhancements among all methods considered, indicating a strong potential for modifying the biopharmaceutical properties of polyphenols. Furthermore, for the described inclusion complexes, the rate of reaching T_max_ remained virtually unchanged when compared to the initial compounds. These modifications exhibited the most pronounced improvement in solubility and bioavailability compared to all the methods studied, as indicated by the highest average AUC value across all modifications.

Another interesting set of results emerged from analyzing publications discussing various phospholipid complexes with flavonoids. There are few methods (Kuche et al., 2019) to prepare these structures for improve biopharmaceutical parameters of different APIs and plant extracts: solvent evaporation (Qiu et al., 2021; Liu et al., 2023), co-grinding (Wang et al., 2020), mechanical dispersion (Patil et al., 2024), the supercritical fluid process, co-solvent lyophilization, and anti-solvent precipitation (Saini et al., 2022). Although the increase in solubility achieved through this method was less than that attained with other approaches, its effect on pharmacokinetic parameters was quite pronounced. The average increase in AUC reached 3.2 times, falling short of the nanostructure and inclusion complex methods (3.7 and 4.2 times, respectively) while being approximately equal to that of micelles. The C_max_ increased by 3.0 times, which ranked just behind micelles and nanostructures (5.4 times, respectively). Additionally, the relative increase in T_1/2_ was the largest among all the methods described for enhancing bioavailability and solubility, reaching 2.1 times. A decrease in T_max_ of nearly 2-fold was also observed. This positions this method as promising for the development of dosage forms that require prolonged release of the active substance based on flavonoids. The similar effect of phospholipid complexes enabled the development of a sustained-release formulation of dehydroandrographolide succinate for the treatment of respiratory diseases (Chen et al., 2025).

The results of the bioavailability assessment of various nanostructures showed the expected results. A significant increase in AUC and C_max_ (3.7 and 5.4 times, respectively) was observed, which is attributed to the increased surface area of the ​​nanostructures compared to other objects. This enhancement leads to a greater completeness of absorption of the active substance and accelerates the process: T_max_ decreased by more than 2 times compared to the initial flavonoids. Thus, it can be concluded that various nanostructures may be a preferable choice for developing fast-acting drugs with high bioavailability. This was exemplified by albendazole, an antiparasitic agent, where solid lipid nanoparticles demonstrated a very rapid release of the active substance, as well as a significant increase in permeability into echinococcal cysts (Movahedi et al., 2017). The effects of increased bioavailability when obtaining nanostructured materials are also manifested at the molecular-genetic level. For instance, nanofiber frames made of polyvinyl alcohol filled with flaxseed extract exhibited a significant increase in the expression of genes that are osteogenic markers (Abdelaziz et al., 2024). This effect of nanostructures can be explained by the reduction in the size of the active substance particles during their production (Leuenberger, 2002). This reduction leads to a larger surface area and results in the aforementioned changes in solubility, bioavailability, and the biological effects of nanostructured forms. In our study, this modification method demonstrated the most pronounced increase in C_max_, while the average AUC ranked second, following the inclusion complex. Furthermore, the major decrease in T_max_ was observed among all the methods analyzed.

Despite the fact that phytosomes are one of the types of phospholipid complexes, this review has chosen to categorize them as a separate group due to their unique characteristics, which involve covalent or hydrogen bonding between the shell and the encapsulated phytocomponent (Kuche et al., 2019; Lu et al., 2019). Because of this feature, these structures did not show a substantial increase in several pharmacokinetic parameters: AUC, C_max_, T_1/2_ (1.7, 1.6, and 1.1 times, respectively). However, these modifications showed the greatest increase in T_max_, suggesting potential for developing dosage forms with prolonged release of the active substance. Moreover, this modification method has recently been the subject of active research for the treatment of various types of cancer (Banerjee et al., 2025; Zhao et al., 2025). Unfortunately, the number of studies investigating the use of this method for individual flavonoids has been insufficient to fully evaluate their impact on the parameters under study. A significant number of publications have focused on researching medicinal plant extracts and the production of phytosomes derived from them.

The method of lyophilization without the addition of excipients to enhance the solubility and bioavailability of flavonoids has proven to be unpopular. This method is widely used to improve the solubility and bioavailability of various APIs (Taldaev et al., 2023). One of the factors contributing to alterations in solubility and pharmacokinetic parameters is the change in the morphology of the substance and the increase in surface area during lyophilization. Moreover, this method is utilized for the production of various nanostructured materials and liposomal forms for drug delivery (Fonte et al., 2016; Franzé et al., 2018).

The method of obtaining micelles has been employed quite infrequently. However, several articles have focused not on solubility, but rather on permeability (Zhang Z. et al., 2017; Shen et al., 2019). The differing polarities within micellar structures facilitate the incorporation of poorly soluble molecules, thereby enhancing their solubilization and increasing the solubility of such compounds (Vinarov et al., 2018). It is important to note that the formation of micellar and phospholipid complexes, as well as phytosomes, are similar processes from a physical chemistry perspective. Nevertheless, we have chosen to categorize them into three distinct groups to maintain the established terminology used by the authors of the included articles.

One of the primary obstacles to systematizing information on methods for enhancing the solubility of various flavonoid modifications lies in the large differences in the methodologies employed in these analyses. Specifically, the variables such as temperature, pH value, dissolution medium, analysis duration, and methods of quantitative determination exhibited considerable variation. Heterogeneity in the conditions and methods used for solubility analysis could led to influence the final outcome of determining the solubility of the resulting objects. This variability hindered the possibility of directly comparing results across different studies and necessitated the exclusion of publications that did not provide information on the solubility of the original compound or the solubility enhancement factor. Consequently, it seems essential to standardize the solubility determination method, for instance, utilizing high-performance liquid chromatography. Moreover, there is a paucity of research dedicated to examining the permeability of the modified flavonoids, which limits the potential for comparing the methods described in this article. The main challenge is that most studies have employed four established methods (cocrystallization, the formation of phospholipid and inclusion complexes, and the generation of nanostructures), leaving relatively little information available on other techniques.

Despite these challenges, we have compiled and summarized the literature regarding methods for enhancing the solubility and bioavailability of flavonoids, and have assessed the comparative effects of various techniques on the pharmacokinetic parameters of the resultant compounds. This information will prove valuable for scientists investigating the bioavailability of this group of natural compounds.

## 5 Conclusion

This review was conducted to summarize and systematize scientific data regarding the enhancement of solubility and bioavailability of flavonoids without changing their molecular structure. Throughout the investigation, it was determined that the most prevalent methods for increasing solubility and bioavailability include co-crystallization, formation of phospholipid and inclusion complexes, and the creation of nanostructures. Although there were no pronounced differences observed in enhancing solubility, the impact of these methods on pharmacokinetic parameters was established. Notably, the greatest average increase in AUC was recorded for various complexes, micelles, and nanostructures, which are the most promising for further study in the field of increasing the bioavailability of flavonoids. The most effective methods for increasing the C_max_ of the active substance in blood plasma were identified as nanostructured forms and micelles. No pronounced effect of the methods on excretion processes was established, while the rate of achieving the maximum concentration of drugs decreased for nanostructures and phospholipid complexes. During the systematization and generalization of the data, a high level of heterogeneity in solubility assessment methods across various studies was revealed, complicating comparisons of original data obtained by different researchers. Taking this into account, it would be beneficial to conduct a comparative analysis of the methods and conditions used to assess solubility in order to identify the most universal parameters for determining solubility across various flavonoids. It is also possible that similar discrepancies may be observed in the case of other groups of compounds, which necessitates further investigation. Another potential avenue for future scientific research in this field is to investigate the feasibility of integrating various methods to produce products with specific biopharmaceutical parameters. The findings of this review are crucial for researchers investigating the bioavailability of flavonoid compounds and will facilitate the selection of the most effective methods based on the desired outcomes for solubility and bioavailability.
