# Supplementary material for: Modification of biopharmaceutical parameters of flavonoids: a review
Source: Front Chem. 2025 Apr 29;13:1602967. doi: 10.3389/fchem.2025.1602967 (PMC12069051; doi:10.3389/fchem.2025.1602967)
Supplement: Supplementary file 1 [file Table1.docx]

Supplementary Material

# Supplementary Tables

**Supplementary Table 1.** Solubility data extracted from included articles.

| Group of flavonoids | Compound | Method of increasing solubility | Conditions for determining solubility | | | | Initial solubility, μg/mL | Resulting solubility, μg/mL | Multiplicity of solubility increase | Reference |
| --- | --- | --- | --- | --- | --- | --- | --- | --- | --- | --- |
|  |  |  | Time, h | pH | t, °C | Dissolution medium |  |  |  |  |
| Isoflavones | Genistein (GEN) | Cocrystallization with piperazine | 10 | 7.0 | 37 | W | 5.10 | 29.31 | 5.75 | (Wang et al., 2023) |
|  |  | Solid dispersions with PVP K30 | 24 | 6.8 | 37 | Phosphate buffer | 1.80 ± 0.10 | 141.30 ± 8.50  (1:3 ratio) | 78.50 | (Qiu et al., 2024) |
|  |  |  |  |  |  |  |  | 768.10 ± 107.90 (1:5 ratio) | 426.72 |  |
|  |  |  |  |  |  |  |  | 1846.60 ± 97.80 (1:7 ratio) | 1025.89 |  |
|  |  |  |  |  |  |  |  | 3981.50 ± 139.60 (1:9 ratio) | 2211.94 |  |
|  |  | Inclusion complex with  HP-β-CD and Poloxamer 188  (binary: GEN+ HP-β-CD; ternary: GEN+ HP-β-CD+ PL 188) | 72 | – | 37 ± 0.5 | W | 2.09 | 10.34 ± 2.25 (binary physical mixture) | 4.95* | (Zafar et al., 2021) |
|  |  |  |  |  |  |  |  | 14.86 ± 3.43 (ternary physical mixture) | 7.11* |  |
|  |  |  |  |  |  |  |  | 81.54 ± 5.87 (binary inclusion complex) | 39.01* |  |
|  |  |  |  |  |  |  |  | 125.76 ± 6.75 (ternary inclusion complex) | 60.17* |  |
|  |  | Cocrystallization with lysine (LYS) and arginine (ARG) | 2 | – | 25 | W | 0.05 | 1191.00 ± 161.00 (GEN – LYS) | 23 820.00* | (Garbiec et al., 2023) |
|  |  |  |  |  |  |  |  | 938.00 ± 14.00 (GEN – ARG) | 18 760.00* |  |
|  | Daidzein (DDZ) | Inclusion complex with  γ-CD (DDZ/γ-CD) and γ-CD-based metal–organic frameworks (DDZ/CD-MOF-1) | 4 | – | 37 ± 0.5 | W | 4.20 ± 0.71 | 3.70 ± 0.16 (DDZ/γ-CD) | 0.88* | (Inoue et al., 2022) |
|  |  |  |  |  |  |  |  | 3.17 × 10^3^ ± 45.60 (DDZ/CD-MOF-1 physical mixture) | 754.76* |  |
|  |  |  |  |  |  |  |  | 4.69 × 10^3^ ± 75.50 (DDZ/CD-MOF-1) | 1116.67* |  |
|  |  |  | 72 | – | 37 ± 0.5 | W | 2.68 ± 0.14 | 4.94 ± 0.15 (DDZ/γ-CD) | 1.84* |  |
|  |  |  |  |  |  |  |  | 1.81 × 10^3^ ± 54.00 (DDZ/CD-MOF-1 physical mixture) | 675.37* |  |
|  |  |  |  |  |  |  |  | 4.39 × 10^3^ ± 114.00  (DDZ/CD-MOF-1) | 1638.06* |  |
|  |  | Cocrystallization with isonicotinamide (INM), theobromine (TB) and cytosine (CYT) | 4 | 6.8 | 37 | Phosphate buffer | 14.09 ± 0.03 | 29.69 ± 0.05  (DDZ – INM) | 2.11 | (Bhalla et al., 2019) |
|  |  |  |  |  |  |  |  | 26.97 ± 0.02 (DDZ – CYT) | 1.91 |  |
|  |  |  |  |  |  |  |  | 24.84 ± 0.03  (DDZ – TB) | 1.76 |  |
|  |  | Inclusion complex with modified β-CD: mono-6-amino-6-deoxy-β-cyclodextrin (NCD) and mono-6-ethylenediamino-6-deoxy-β-cyclodextrin (ENCD) | 1 | 7.0 | 25 | W | 8.31 | 15 200.00  (DDZ – NCD) | 1829.12* | (Deng et al., 2017) |
|  |  |  |  |  |  |  |  | 13 200.00 (DDZ – ENCD) | 1588.45* |  |
|  |  | Cocrystallization with piperazine | 48 | 7.0 | 37 | W | 4.86 ± 1.25 | 46.80 ± 2.52 (physical mixture) | 9.63* | (Wang et al., 2024) |
|  |  |  |  |  |  |  |  | 295.75 ± 21.80 (cocrystal) | 60.85 |  |
|  |  | Lipid nanocarriers | – | – | – | W | 2.00 | 2000.00 | 1000 | (Zhang et al., 2011) |
| Flavonols | Fisetin | Nanoparticles with PVP | 24 | 7.4 | 37 | PBS | 31.50 | 63.00 | 2.00 | (Chen et al., 2020) |
|  |  | Inclusion complex with cycloamylose | – | – | – | W | 10.00 | 37.07 × 10^3^ ± 581.00 | 3707.00 | (Jeong et al., 2023) |
|  | Quercetin | Nanoparticles (evaporative precipitation of nanosuspension) | 2 | – | 37 ± 0.5 | W | – | – | 7.69 | (Kakran et al., 2011) |
|  |  | Inclusion complex with  β-CD |  |  |  |  |  | – (physical mixture 1:1) | 1.62 |  |
|  |  |  |  |  |  |  |  | –  (complex 1:1) | 7.83 |  |
|  |  |  |  |  |  |  |  | – (complex 1:2) | 8.76 |  |
|  |  |  |  |  |  |  |  | – (complex 1:4) | 9.85 |  |
|  |  | Solid dispersions with PVP |  |  |  |  |  | – (physical mixture 1:1) | 1.44 |  |
|  |  |  |  |  |  |  |  | – (solid dispersion 1:1) | 9.38 |  |
|  |  |  |  |  |  |  |  | – (solid dispersion 1:2) | 9.89 |  |
|  |  |  |  |  |  |  |  | – (solid dispersion 1:4) | 10.20 |  |
|  |  | Solid dispersions with pluronic F127 |  |  |  |  |  | – (physical mixture 1:1) | 1.44 |  |
|  |  |  |  |  |  |  |  | – (solid dispersion 1:1) | 9.28 |  |
|  |  |  |  |  |  |  |  | – (solid dispersion 1:2) | 9.89 |  |
|  |  |  |  |  |  |  |  | – (solid dispersion 1:4) | 10.30 |  |
|  |  | Phospholipid complex | 24 | – | RT | W | 3.44 | 36.81 | 10.70 | (Singh et al., 2012) |
|  | Morin (MOR) | Inclusion complex with  HP-β-CD and SBE- β-CD | 48 | – | 25.0 ± 0.5 | W | – | – (MOR – HP-β-CD) | 100.00 | (De Gaetano et al., 2023) |
|  |  |  |  |  |  |  |  | – (MOR – SBE-β-CD) | 115.00 |  |
|  |  | Inclusion complex with  HP-β-CD | 4 | – | 37 ± 0.5 | 0.1% Tween-80 | 2.80 × 10^3^* | 9.78 × 10^3^* | 3.49* | (Lima et al., 2019) |
|  |  | Nanosuspension (ultrasonic-nanoprecipitation technique) | 12 | – | RT | W | 28.80 ± 2.20 | 127.11 ± 3.20 | 4.41 | (Jangid et al., 2020) |
|  | Myricetin (MYR) | Cocrystallization with proline (PRO), nicotinamide (NIC), isonicotinamide (INM), and caffeine (CAF) | 12 | – | 37 ± 0.5 | W | 16.60 | 63.93 ± 10.71 (MYR – PRO) | 3.85* | (Ren et al., 2019) |
|  |  |  |  |  |  |  |  | 28.30 ± 0.38 (MYR–NIC) | 1.70* |  |
|  |  |  |  |  |  |  |  | 26.07 ± 0.25 (MYR– INM) | 1.57* |  |
|  |  |  |  |  |  |  |  | 8.08 ± 0.22 (MYR– CAF) | 0.49* |  |
|  |  | Nanofibers with HP-β-CD and PVP in different rations (MYR:HP-β-CD:PVP) | 0.17 | – | – | W | 0.32 ± 0.16 | 852.83 ± 21.38 (1:20:4 ratio) | 2665.09* | (Lin et al., 2023) |
|  |  |  |  |  |  |  |  | 892.01 ± 10.11 (1:20:8 ratio) | 2787.53* |  |
|  |  |  |  |  |  |  |  | 914.67 ± 13.18 (1:20:12 ratio) | 2858.32* |  |
|  | Isorhamnetin | Phospholipid complex | – | – | – | W | – | – | 122.00 | (Zou et al., 2022) |
|  | Kaempferol | Phospholipid complex | 36 | – | 25 | W | 1.34 | 290.38 | 216.70* | (Zhang et al., 2015) |
| Flavanones | Naringenin (NAR) | Surfactant-stabilized nanosuspension | 24 | 7.0 | RT | W | 11.20 ± 3.10 | 114.10 ± 4.70 | 10.19* | (Singh et al., 2018) |
|  |  | Phytosomes with LS-75 | 24 | – | RT | W | 24.65 ± 0.46 | 27.24 ± 0.26 (physical mixture) | 1.11* | (Metkari et al., 2023) |
|  |  |  |  |  |  |  |  | 176.55 ± 0.25 (phytosome) | 7.16* |  |
|  |  | Cocrystallization with nicotinamide (NIC) and caffeine (CAF) | 1.5 | 6.8 | 37.0 ± 0.5 | Phosphate buffer | 18.09 | 35.05  (NAR – NIC) | 1.94* | (Cui et al., 2019) |
|  |  |  |  |  |  |  |  | 49.13 (NAR – CAF) | 2.72* |  |
|  | Hesperetin (HES) | Inclusion complex with  β-CD and HP-β-CD | 1 | 6.0 | 20 ± 2 | W | 1.36 | 290.00 (HES – β-CD) | 213.24* | (Yang et al., 2016) |
|  |  |  |  |  |  |  |  | 560.00 (HES – HP-β-CD) | 411.76* |  |
|  |  | Cocrystallization with piperine | 12 | 6.8 | 37 ± 0.2 | – | 21.12 | 41.55 | 1.97* | (Liu et al., 2022) |
| Flavanonols | Dihydroquercetin (DHQ) | Lyophilization from ethanol and acetonitrile solutions | – | – | – | W | 700.00 ± 20.00 | 3090.00 ± 90.00 (from ethanol sol.) | 4.41* | (Terekhov et al., 2022) |
|  |  |  |  |  |  |  |  | 2140.00 ± 60.00  (from acetonitrile sol.) | 3.06 |  |
|  |  | Inclusion complex with  β-CD | – | – | 25 ± 1 | PBS | 520.27 ± 8.68* | 11 771.43 ± 441.46* | 22.63 | (Xu et al., 2023) |
|  |  | Inclusion complex with  β-CD and lecithin | – | – | 25 ± 1 | W | 240.00 ± 10.00 | 5980.00 ± 20.00 (DHQ–β-CD) | 24.92* | (Zhang et al., 2017a) |
|  |  |  |  |  |  |  |  | 1240.00 ± 20.00 (DHQ– lecithin) | 5.17* |  |
| Flavones | Baicalein (BA) | Phospholipid complex (BaPC), matrix dispersion based on phospholipid complex (BaPC-MD), and physical mixture of BaPC/PVP | – | – | 25 | W | 17.50 ± 0.08 | 970.36 ± 12.47 (BaPC) | 55.45* | (Zhou et al., 2017) |
|  |  |  |  |  |  |  |  | 1191.44 ± 4.78 (physical mixture) | 68.08* |  |
|  |  |  |  |  |  |  |  | 1510.04 ± 15.14 (BaPC-MD) | 86.29* |  |
|  |  | Baicalein-solubilizing glycyrrhizic acid nano-micelles | 6 | – | 37 | W | 0.15 ± 0.01 | 690.90 ± 2.69 | 4606.00* | (You et al., 2021) |
|  | Luteolin (LUT) | Cocrystallization with isoniazid (ISN) and caffeine (CAF) | 24 | – | 25 | 0.2% SDS | 35.10 | 112.30 (LUT – ISN) | 3.20 | (Luo et al., 2019) |
|  |  |  |  |  |  |  |  | – (LUT – CAF) | 2.10 |  |
|  |  | Phospholipid complex | 24 | – | RT | W | 930.00 ± 28.00 | 2360.00 ± 89.00 | 2.54* | (Khan et al., 2014) |
|  |  | Nanoparticles (anti-solvent precipitation) and physical mixture of LUT with γ-CD and PVP-k30 | 48 | – | 37 | W | 1.09 ± 0.16 | 15.51 ± 1.31 (physical mixture) | 14.23* | (Wang et al., 2019) |
|  |  |  |  |  |  |  |  | 149.69 ± 12.6 (LUT nanoparticles) | 132.74 |  |
|  | Apigenin (AP) | Inclusion complex with  HP-β-CD | 48 | 6.8 | 37 | – | 0.17 | 0.28  (micronized AP) | 1.65* | (Wu et al., 2017b) |
|  |  |  |  |  |  |  |  | 6.81 (AP – HP-β-CD) | 40.06* |  |
|  |  | Phospholipid phytosome | 24 | – | 25 | W | 0.62 ± 0.88 | 6.13 ± 1.13 (physical mixture) | 9.89* | (Telange et al., 2017) |
|  |  |  |  |  |  |  |  | 22.80 ± 1.40 (phytosome) | 36.77* |  |
|  |  | Nanoparticles with mannitol | 48 | 6.8 | 37 | – | 0.81 | 15.57 (physical mixture) | 19.22* | (Wu et al., 2017a) |
|  |  |  |  |  |  |  |  | 52.50 (nanoparticles) | 64.81 |  |
|  | Chrysin (CHR) | Inclusion complex with random methyl-β-cyclodextrin (RAMEB), SBE- β-CD, HP-β-CD, and β-CD | 24 | – | RT | W | 1.01 ± 0.07 | 7.48 ± 0.15 (1:1 with RAMEB) | 7.41 | (Fenyvesi et al., 2020) |
|  |  |  |  |  |  |  |  | 8.12 ± 0.42  (1:2 with RAMEB) | 8.04 |  |
|  |  |  |  |  |  |  |  | 6.35 ± 0.11 (1:1 with SBE- β-CD) | 6.29 |  |
|  |  |  |  |  |  |  |  | 7.32 ± 0.20 (1:2 with SBE- β-CD) | 7.25 |  |
|  |  |  |  |  |  |  |  | 5.72 ±0.28 (1:1 with HP-β-CD) | 5.66 |  |
|  |  |  |  |  |  |  |  | 7.59 ± 0.17 (1:2 with HP-β-CD) | 7.52 |  |
|  |  |  |  |  |  |  |  | 4.42 ±0.37 (1:1 with β-CD) | 4.37 |  |
|  |  | Cocrystallization with cytosine (CYT) and thiamine hydrochloride (THI) | 24 | 6.8 | 37+0.5 | Phosphate buffer | 6.1 | 13.7 (CHR – CYT) | 2.25* | (Chadha et al., 2017) |
|  |  |  |  |  |  |  |  | 24.8 (CHR – THI) | 4.07* |  |
|  | Scutellarein | Inclusion complex with  HP-β-CD | 1 | 7.0 | 25 ± 2 | W | 8.98 | 4020.00 | 447.66* | (Wang et al., 2014) |

* values were calculated by the authors of current review
(PVP – polyvinylpyrrolidone; CD – cyclodextrin; HP-β-CD – 2-hydroxypropyl beta-cyclodextrin; SBE- β-CD – sulfobutylether
β-cyclodextrin; W – water; PBS – phosphate-buffered saline; SDS – sodium dodecyl sulfate)
