# Supplementary material for: Modification of biopharmaceutical parameters of flavonoids: a review
Source: Front Chem. 2025 Apr 29;13:1602967. doi: 10.3389/fchem.2025.1602967 (PMC12069051; doi:10.3389/fchem.2025.1602967)
Supplement: Supplementary file 2 [file Table2.docx]

Supplementary Material

# Supplementary Tables

**Supplementary Table 2.** Pharmacokinetic parameters extracted from included articles.

| Group | Compound | Method of increasing bioavailability | Analyzed sample | Dose, mg/kg | Pharmacokinetic parameters | | | | Reference |
| --- | --- | --- | --- | --- | --- | --- | --- | --- | --- |
|  |  |  |  |  | AUC, μg/mL*h | T_max_, h | C_max_, μg/mL | T_1/2_, h |  |
| Isoflavones | Genistein (GEN) | Cocrystallization with piperazine | GEN | 100 | 2.36 ± 0.27 | 5.67 ± 0.82 | 0.31 ± 0.09 | 4.02 ± 1.71 | (Wang et al., 2023) |
|  |  |  | Cocrystal |  | 3.79 ± 0.81 | 7.33 ± 1.03 | 0.41 ± 0.11 | 5.58 ± 1.94 |  |
|  |  | Solid dispersions with PVP K30 | GEN | 50 | 5.20 ± 0.80 | 4.70 ± 0.90 | 0.60 ± 0.10 | 6.70 ± 0.20 | (Qiu et al., 2024) |
|  |  |  | Solid dispersion 1:7 |  | 10.70 ± 1.60 | 4.40 ± 0.50 | 0.80 ± 0.10 | 4.50 ± 1.40 |  |
|  |  | Micelle formation | GEN | 60 | 5.23×10^-3^ ± 0.62×10^-3^ | 0.96 ± 0.10 | 0.69×10^-3^ ± 0.06×10^-3^ | 3.93 ± 0.93 | (Shen et al., 2018) |
|  |  |  | Micelle |  | 12.69×10^-3^ ± 1.38×10^-3^ | 0.71 ± 0.19 | 3.53×10^-3^ ± 0.20×10^-3^ | 3.23 ± 0.88 |  |
|  |  | Micelle  formation | GEN | 60 | 3.35 ± 1.18 | 0.62 ± 0.14 | 0.44 ± 0.14 | 4.64 ± 1.99 | (Ding et al., 2019) |
|  |  |  | Micelle (1.2 mg/ml) |  | 10.82 ± 1.54 | 0.67 ± 0.13 | 2.40 ± 0.48 | 3.30 ± 1.16 |  |
|  |  |  | Micelle (1.6 mg/ml) |  | 14.93 ± 5.58 | 0.71 ± 0.10 | 3.56 ± 1.12 | 2.79 ± 0.91 |  |
|  | Daidzein (DDZ) | Cocrystallization with  isonicotinamide (INM),  theobromine (TB), cytosine (CYT) | DDZ | 10 | 1.28 | 4.00 | 0.87 ± 0.02×10^-3^ | – | (Bhalla et al., 2019) |
|  |  |  | Cocrystal DDZ – INM |  | 2.70 | 3.00 | 1.85 ± 0.05×10^-3^ | – |  |
|  |  |  | Cocrystal DDZ – TB |  | 2.20 | 3.00 | 1.33 ± 0.02×10^-3^ | – |  |
|  |  |  | Cocrystal DDZ – CYT |  | 2.45 | 3.00 | 1.61 ± 0.03×10^-3^ | – |  |
|  |  | Cocrystallization with piperazine | DDZ | 100 | 0.90 ± 0.39 | 3.75 ± 2.89 | 0.13 ± 0.07 | 8.58 ± 6.16 | (Wang et al., 2024) |
|  |  |  | Cocrystal |  | 2.82 ± 0.76 | 8.17 ± 2.71 | 0.27 ± 0.05 | 4.76 ± 2.18 |  |
|  |  | Lipid nanocarriers | DDZ | 20 | 6.78 ± 0.47 | 3.00 | 0.67 ± 0.07 | – | (Zhang et al., 2011) |
|  |  |  | Phospholipid complex |  | 24.54 ± 2.66 | 0.42 | 6.83 ± 1.11 | – |  |
|  |  |  | Lipid nanocarriers |  | 46.58 ± 6.22 | 0.25 | 14.51 ± 2.39 | – |  |
| Flavonols | Morin (MOR) | Inclusion complex with HP-β-CD | MOR | 100 | 1.41 | 4.00 | 0.22 ± 4.7×10^-3^ | – | (Lima et al., 2019) |
|  |  |  | Inclusion complex |  | 5.93 | 1.50 | 0.68 ± 1.2×10^-3^ | – |  |
|  |  | Nanosuspension | MOR | 50 | 1.47 | – | 0.58 | – | (Jangid et al., 2020) |
|  |  |  | Nano-suspension |  | 3.00 | – | 0.89 | – |  |
|  | Kaempferol | Phospholipid complex | Kaempferol | 6 | 17.72 ± 2.82 | 5.00 ± 1.01 | 1.43 ± 0.21 | 5.11 ± 1.13 | (Zhang et al., 2015) |
|  |  |  | Phospholipid complex |  | 59.23 ± 10.08 | 5.17 ± 1.32 | 3.94 ± 0.83 | 10.51 ± 1.05 |  |
| Flavanones | Naringenin (NAR) | Surfactant-stabilized nanosuspension | NAR | 30 | 2.23 | – | 0.34 ± 0.09 | 5.32 ± 1.27 | (Singh et al., 2018) |
|  |  |  | Nano-suspension |  | 8.40 | – | 0.73 ± 0.15 | 5.50 ± 1.95 |  |
|  |  | Phytosomes with  LS-75 | NAR | 46.5 | 13.89 ± 0.74 | 2.00 ± 0.26 | 1.43 ± 0.13 | 8.21 ± 0.22 | (Metkari et al., 2023) |
|  |  |  | Phytosomes |  | 26.44 ± 0.87 | 1.50 ± 0.12 | 2.53 ± 0.26 | 10.09 ± 0.27 |  |
|  |  | Cocrystallization with nicotinamide (NIC) and caffeine (CAF) | NAR | 100 | 2.93 ± 0.90 | 2.20 ± 1.10 | 0.51 ± 0.31 | – | (Cui et al., 2019) |
|  |  |  | Cocrystal NAR – NIC |  | 2.37 ± 0.47 | 0.08 ± 0.00 | 2.91 ± 1.61 | – |  |
|  |  |  | Cocrystal NAR – CAF |  | 4.14 ± 1.51 | 0.40 ± 0.34 | 1.65 ± 0.88 | – |  |
|  | Hesperetin (HES) | Cocrystallization with piperine (PIP) | HES | 80 | 0.53 | 0.50 | 0.12 | 3.01 | (Liu et al., 2022) |
|  |  |  | Cocrystal HES – PIP |  | 3.23 | 1.00 | 0.61 | 2.68 |  |
| Flavones | Baicalein (BA) | Phospholipid complex (BaPC), matrix dispersion based on phospholipid complex (BaPC-MD) | BA | 75 | 12.01 ± 4.73 | 2.83 ± 1.10 | 1.61 ± 0.37 | – | (Zhou et al., 2017) |
|  |  |  | BaPC |  | 37.13 ± 12.56 | 0.56 ± 0.14 | 8.68 ± 1.35 | – |  |
|  |  |  | BaPC-MD |  | 103.64 ± 26.02 | 0.81 ± 0.14 | 12.75 ± 1.77 | – |  |
|  |  | Micelle formation | BA | 40 | 40.99 ± 10.23 | 1.34 ± 0.45 | 1.38 ± 1.23 | 3.45 ± 0.33 | (Shen et al., 2019) |
|  |  |  | Micelle |  | 123.79 ± 28.45 | 1.56 ± 0.34 | 6.32 ± 1.92 | 4.89 ± 0.22 |  |
|  | Luteolin (LUT) | Cocrystallization with isoniazid (ISN) and caffeine (CAF) | LUT | – | 3.06 ± 1.03 | 0.50 ± 0.00 | 0.72 ± 0.10 | 5.60 ± 1.00 | (Luo et al., 2019) |
|  |  |  | Cocrystal LUT – ISN |  | 8.13 ± 2.39 | 0.50 ± 0.00 | 1.53 ± 0.13 | 5.20 ± 0.90 |  |
|  |  |  | Cocrystal LUT – CAF |  | 4.42 ± 1.44 | 0.50 ± 0.00 | 0.93 ± 0.25 | 5.40 ± 0.70 |  |
|  |  | Nanoparticles  (anti-solvent precipitation) | LUT | 30 | 9.73×10^-3^ ± 0.17×10^-3^ | 0.25 ± 0.02 | 0.53 ± 0.01 | 45.40 ± 3.13 | (Wang et al., 2019) |
|  |  |  | Nanoparticles |  | 20.85×10^-3^ ± 0.21×10^-3^ | 0.25 ± 0.01 | 0.89 ± 0.02 | 36.96 ± 3.01 |  |
|  | Apigenin (AP) | Inclusion complex with HP-β-CD | AP | 60 | – | 2.00 | 0.04 | – | (Wu et al., 2017b) |
|  |  |  | Inclusion complex |  | – | 3.00 | 0.12 | – |  |
|  |  | Phospholipid phytosome | AP | 100 | 1.27 ± 0.28 | 2.00 ± 0.23 | 0.14 ± 0.15 | 4.80 ± 0.33 | (Telange et al., 2017) |
|  |  |  | Phytosome |  | 1.95 ± 0.65 | 4.00 ± 0.34 | 0.20 ± 0.25 | 4.34 ± 0.52 |  |
|  |  | Nanoparticles with mannitol | AP | 50 | – | 2.00 | 12.69×10^-3^ | – | (Wu et al., 2017a) |
|  |  |  | Nanoparticles |  | – | 0.33 | 1.39×10^-3^ | – |  |
|  |  | Micelle formation | AP | 40 | 9.57 ± 3.09 | 2.00 | 1.86 ± 0.54 | 2.50 ± 1.01 | (Zhang et al., 2017b) |
|  |  |  | Micelle |  | 32.93 ± 6.07 | 2.00 | 6.65 ± 1.33 | 1.75 ± 0.22 |  |
|  | Chrysin (CHR) | Cocrystallization with cytosine (CYT) and thiamine hydrochloride (THI) | CHR | 100 | 51.78×10^-3^ | – | 17.56×10^-3^ | – | (Chadha et al., 2017) |
|  |  |  | Cocrystal CHR – CYT |  | 84.96×10^-3^ | – | 28.93×10^-3^ | – |  |
|  |  |  | Cocrystal CHR – THI |  | 100.62×10^-3^ | – | 34.26×10^-3^ | – |  |

(PVP – polyvinylpyrrolidone; HP-β-CD – 2-hydroxypropyl beta-cyclodextrin)
